# Spatiotemporal analysis of *highly pathogenic avian influenza* (H5N1) outbreaks in poultry in Egypt (2006 to 2017)

**DOI:** 10.1186/s12917-022-03273-w

**Published:** 2022-05-12

**Authors:** Yumna Elsobky, Gamal El Afandi, Akram Salama, Ahmed Byomi, Muhammad Omar, Mahmoud Eltholth

**Affiliations:** 1 Department of Hygiene and Zoonosis, Faculty of Veterinary Medicine, University of Sadat City, Menofia, Sadat City, 32897 Egypt; 2 College of Agriculture, Environment and Nutrition Sciences, Tuskegee University, Tuskegee, AL USA; 3 Astronomy and Meteorology Department, Faculty of Science, Al-Azhar University, Cairo, Egypt; 4 Department of Animal Medicine and Infectious Diseases, Faculty of Veterinary Medicine, University of Sadat City, Sadat City, Menofia 32897 Egypt; 5 Department of Biomedical Sciences, College of Veterinary Medicine, Tuskegee University, Tuskegee, AL USA; 6 Global Academy of Agriculture and Food Security, The Royal (Dick) School of Veterinary Studies, University of Edinburgh, Easter Bush Campus, Midlothian, EH25 9RG UK; 7 Department of Animal Hygiene and Preventive Medicine, Faculty of Veterinary Medicine, Kafrelsheikh University, Kafrelsheikh, Egypt

**Keywords:** Egypt, HPAI H5N1, Epidemic wave, Spatiotemporal pattern, Spatial analysis, Clusters epidemics

## Abstract

**Background:**

In Egypt, the highly pathogenic avian influenza (HPAI) subtype H5N1 is endemic and possesses a severe impact on the poultry. To provide a better understanding of the distributional characteristics of HPAI H5N1 outbreaks in Egypt, this study aimed to explore the spatiotemporal pattern and identify clusters of HPAI H5N1 outbreaks in Egypt from 2006 to 2017.

**Results:**

The Epidemic curve (EC) was constructed through time series analysis; in which six epidemic waves (EWs) were revealed. Outbreaks mainly started in winter peaked in March and ended in summer. However, newly emerged thermostable clades (2.2.1.1 and 2.2.1.2) during the 4th EW enabled the virus to survive and cause infection in warmer months with a clear alteration in the seasonality of the epidemic cycle in the 5th EW. The endemic situation became more complicated by the emergence of new serotypes. As a result, the EC ended up without any specific pattern since the 6th EW to now. The spatial analysis showed that the highest outbreak density was recorded in the Nile Delta considering it as the ‘Hot spot’ region. By the 6th EW, the outbreak extended to include the Nile valley. From spatiotemporal cluster epidemics, clustering in the Delta was a common feature in all EWs with primary clusters consistently detected in the hot-spot region, but the location and size varied with each EW. The highest Relative Risk (RR) regions in an EW were noticed to contain the primary clusters of the next EW and were found to include stopover sites for migratory wild birds. They were in Fayoum, Dakahlia, Qalyobiya, Sharkia, Kafr_Elsheikh, Giza, Behera, Menia, and BeniSuef governorates. Transmission of HPAI H5N1 occurred from one location to another directly resulted in a series of outbreaks forming neighboring secondary clusters. The absence of geographical borders between the governorates in addition to non-restricted movements of poultry and low vaccination and surveillance coverage contributed to the wider spread of infection all over Egypt and to look like one epidemiological unit.

**Conclusion:**

Our findings can help in better understanding of the characteristics of HPAI H5N1 outbreaks and the distribution of outbreak risk, which can be used for effective disease control strategies.

**Graphical abstract:**

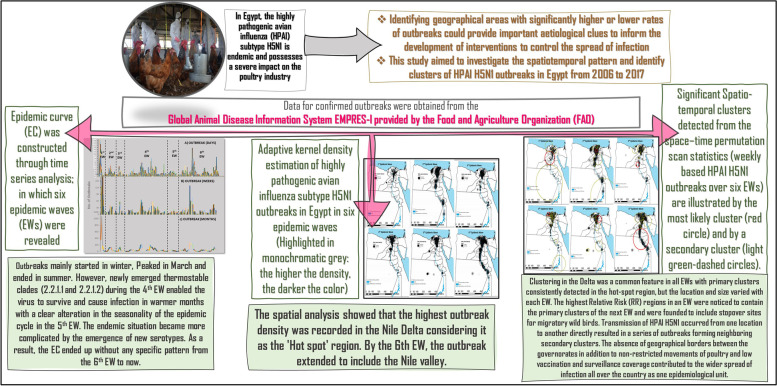

## Introduction

In Egypt, poultry production is one of the fastest-growing agricultural sectors with a very high economic importance [1]. More than 75% of poultry is produced in unregulated small to medium-scale commercial or backyard farms characterized by low-to-no biosecurity measures [1, 2]. Highly pathogenic avian influenza (HPAI) subtype H5N1 clade 2.2 was first detected in Egypt from a Eurasian green-winged teal in Damietta governorate in 2005, this was followed by the detection of genetically closely related HPAI H5N1 viruses in domestic birds and humans in February 2006 [3]. Since then, HPAI H5N1 outbreaks in poultry resulted in severe economic losses for the industry and the livelihood of more than 1.5 million people has been affected [4]. The first wave of the disease resulted in the culling of about 40 million birds, for which the estimated costs of compensation were US$ 29,375,000 [5, 6]. However, this compensation was far less than the actual production costs from farmers’ perspectives and consequently, many farmers and breeders stopped reporting the disease particularly after 2008 when the compensation scheme was also stopped [1, 4, 7]. This resulted in decreasing notifications of the outbreaks and endless circulation of the virus in the poultry population [8, 9].

The Egyptian authorities have made constant efforts to mitigate the disease including increasing public awareness; stamping out infected birds (within 3 km of the initial outbreak); surveillance; banning live bird markets; restricting poultry movement within 7 km radius from the outbreak location and emergency vaccination of parent flocks [5, 10]. These measures failed to limit the spread of infection therefore, the decision was taken to vaccinate all commercial flocks and backyard poultry, surveillance, and preemptive culling of infected birds [1, 5]. Different types of surveillance programs (active, passive, and targeted surveillances) were conducted to elucidate the spread of infection in poultry sectors [1]. The National Laboratory for Veterinary Quality Control on Poultry Production (NLQP) was established for all surveillance activities in poultry nationwide. In addition to the international cooperation with the World Organization for Animal Health (World Organization for Animal Health (OIE), #24) reference laboratories through twinning programs and other projects [1].

Despite these control measures, HPAI H5N1 became endemic by 2008 with continuous and extensive circulation revealed by the regular nationwide active, passive, and targeted surveillance activities [1, 8, 9, 11–13]. Vaccination has become the main tool to control the HPAI H5N1 virus in Egypt, as other aspects of the control strategies are neglected, including biosecurity [14]. Globally, Egypt is the second country after China in terms of HPAI H5N1 vaccination usage [15]. However, mass vaccination is not effective without adequate coverage and if not complemented by appropriate outbreak management and bio-security measures [4]. Consequently, the efficacy of vaccination decreased overtime followed by vaccine failures due to the emergence of antigenic drift variants [16]. Egypt has become an epicenter for A(H5) virus evolution, and outbreaks in poultry continued to occur with genetic drift in the hemagglutinin (HA) gene observed each year [4, 17]. This in addition to the structure of the poultry industry were the main challenges to effectively control the spread of infection in Egypt [16].

In Egypt, most genetic changes in the virus circulating in dense poultry populations occurred between 2006 to 2015 [16]. Clades 2.2.1 and 2.2.1.1 had been emerged as a vaccine-escape mutant between 2009 and 2011 due to mutation in HA protein [16, 18, 19]. Clade 2.2.1 continuously evolved to clade 2.2.1.2a which leads to increase human infections [20]. The situation has been worthen by the introduction of Low Pathogenic Avian Influenza (LPAI) H9N2 virus in 2010 [21, 22], and HPAI H5N8 virus in 2017 [23, 24]. Co-circulation of all these serotypes in the poultry population leads to continuous virus evolution that affects virus characteristics [25, 26]. Wild birds can transmit Avian Influenza Viruses to domestic poultry either directly or indirectly via a contaminated environment [27]. Along the Mediterranean coast, Red Sea coast, Nile delta, and Nile Valley locate highly populated wetlands with waterfowl [28, 29]. BirdLife International identifies 34 important bird and biodiversity areas (IBAs) [29], including the four Ramsar sites [30]. Peak prevalence of the most frequent AIV carrier birds occurred during fall migration [31, 32] which can be further transmitted to local domestic birds [2].

Although there is no significant data on HPAI H5N1 for 2018 and 2019 [33], several studies declared a widespread of the virus among poultry flocks in Egypt [34]. Infection in backyard poultry is usually associated with mild or no symptoms [2], calling for the need to develop an efficient surveillance program and investigate the effectiveness of the current implemented control measures [34]. The impact of vaccination on the temporal and spatial distribution of the outbreaks in endemic areas is debated [35]. Although the direct association between vaccination and HPAI H5N1 virus evolution is difficult to establish, vaccination in combination with the culling of infected birds/farms may be the most appropriate way to control infections [35]. The evolutionary patterns and temporal distribution of the virus are important for making targeted vaccination policies and developing appropriate preventive measures [36–38]. It could also help to identify potential areas of subsequent outbreaks around epidemic areas. This will support estimating an appropriate radius for prevention and culling, and establishing early warning systems for regions potentially affected [35]. Therefore, this study aimed to investigate the spatiotemporal pattern and identify clusters of HPAI H5N1 outbreaks in Egypt from 2006 to 2017. The output of this study would provide a better understanding of the distributional characteristics of outbreaks in Egypt and offer prospects for effective disease control strategies.

## Data and methods

### Data sources

HPAI-H5N1 outbreak data werer collected from two sources: the Egyptian ministry of agriculture (General Organization For Veterinary Services) official reports for national surveillance and the Global Animal Disease Information System (EMPRES-I) database available at the Food and Agriculture Organization (FAO) (FAO, [39]). These resulted in 7433 confirmed outbreaks of HPAI H5N1 in domestic poultry were officially reported between January 2006 and December 2017. All data were integrated into one dataset.

An outbreak was defined as “*the confirmed presence of disease, clinically expressed or not, in at least one individual in a defined location and during a specified period*”. The Spatiotemporal attributes of each outbreak, date and the centroids, were used. The country map was constructed by (ArcGIS 10.5 software) to facilitate the presentation of data and the interpretation of results.

### Data analysis

Daily, weekly, and monthly epidemic curves were constructed to display the magnitude and temporal trends of HPAI H5N1 outbreaks all over the country. For each epidemic wave (EW), a number of disease outbreaks peaked rapidly and then decreased gradually until the epidemic was over [40], the number of outbreaks was calculated.

Kernel density estimation (KDE) is a simple non-parametric technique that relies on a few assumptions about the structure of the observed data [41]. It is equivalent to a simple diffusion model that is a useful approximation to patterns of distribution frequently found in ecological data [42]. KDE was used to identify high-density areas [43]; run in Environmental Systems Research Institute ArcMap 10.5 software using reported cases to generate a density surface for each EW.

The quartic kernel function [44, 45] is given by:

$${\hat{\uplambda}}_i\ (S)=\sum_{d_{i\le \tau }}\frac{3}{{\pi \tau}^2}{\left(1-\frac{d_i^2}{\tau^2}\right)}^2$$

where:

1. i = *1*,*…*,*n* are the input points.
2. d_i_ is the distance between the point s and the observed event in location,
3. s_i_ and τ is the radius centered on s.

The formula to calculate the bandwidth is as follows (ESRI, [46]):

$$Search\ Radius=0.9\ast \min \left( SD,\sqrt{\frac{1}{In(2)}}\ast {D}_m\right)\ast {n}^{-0.2}$$

where:

- SD is the standard distance
- D_m_ is the median distance
- n is the number of points if no population field is used, or if a population field is supplied, n is the sum of the population field values

Kernel density maps for the total number of cases were plotted for each EW to visualise the risk for the disease. Default cells and the output were selected in Square kilometer (Km^2^).

Retrospective space-time permutation scan statistics were used to identify the Spatiotemporal clusters for each EW by testing whether outbreaks were correlated in space and time using SaTScan 8.2.1 software [47–49]**.** The scanning window was a cylinder with the spatial and temporal dimensions as circular base and height, respectively. For each EW, the temporal scanning window was set at < 50% of the study period and a maximum of 50% of outbreaks were allowed in the spatial scanning window [50].

The likelihood ratio statistic was used to evaluate the possibility of a true spatiotemporal cluster in a window. The window with the maximum likelihood ratio statistic was considered the primary cluster while the remainder were considered secondary clusters. The statistical significance was tested through Monte Carlo simulations of 999 replications [50]. The time units of a week and a month were used. The results from daily, weekly and monthly outbreaks were very similar, therefore only week-based results were reported.

ArcGIS 10.5 software ESRI, Redlands, California, USA) was used to overlay results from different methods in a map for visual comparisons**.**

## Results

### Epidemic waves of HPAI H5N1 outbreaks in Egypt

**Fig. 1:**
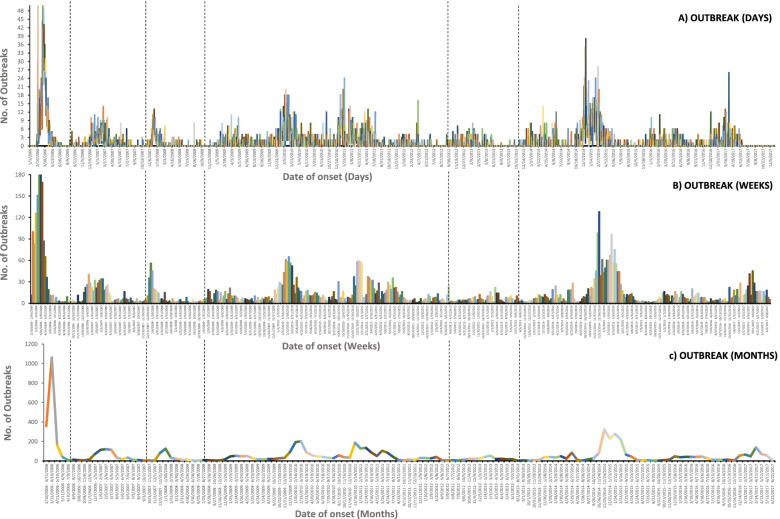
Epidemic curves of outbreaks of highly pathogenic avian influenza subtype H5N1 in Egypt (January 2006 to December 2017), illustrating **A**) daily, **B**) weekly, and **C**) monthly frequency of outbreaks as a function of time. Vertical lines delineate the six epidemic waves

**Fig. 2:**
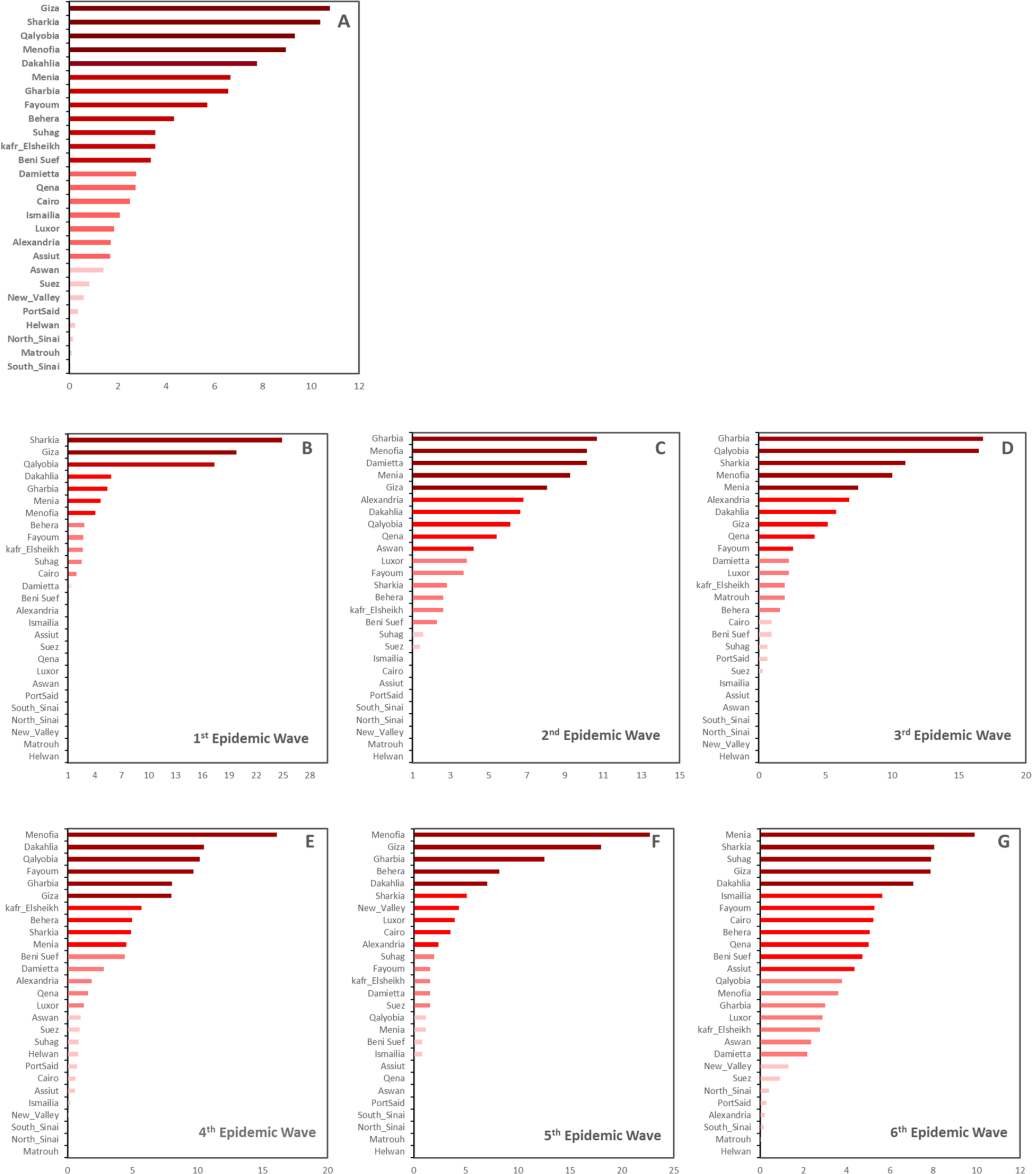
Spatial distribution of HPAI H5N1 outbreaks in Egyptian governorates in the six epidemic waves

In Egypt, six epidemic waves (EW1–6) of HPAI H5N1 outbreaks were identified over the study period, Fig. 1. The 1st EW began in February 2006, peaked in March 2006, and ended in July 2006. This was the first introduction of the disease in Egypt with 1627 outbreaks, Fig. 1. The highest numbers of outbreaks were in Sharkia, Giza, Qalyobiya, Dakahlia, Gharbia, Menia, and Menofia governorates, respectively, Fig. 2A. The 2nd EW began in October 2006, peaked in March 2007, and ended in August 2007; with 571 outbreaks, Fig. 1. Unlike the 1st EW, the highest numbers of outbreaks were in Gharbia, Menofia, Damietta, Menia, Giza, Alexandria, Dakahlia Qalyobiya, Qena, Aswan, and Luxor governorates respectively, Fig. 2B. The 3rd EW began in November 2007, peaked in January 2008, and ended in July 2008; with 309 outbreaks, Fig. 1. The highest numbers of outbreaks were in Gharbia, Qalyobiya, Sharkia, and Menofia governorates respectively, Fig. 2C. The 4th EW began in November 2008 and ended in July 2012 with three successive peaks in March 2009, March 2010, and March 2011. This was the longest EW with three epidemic cycles; from January 2009 to August 2009, January 2010 to August 2010, and from January 2011 to August 2011, respectively. All cycles peaked in March with outbreaks observed all over the year. The total number of recorded outbreaks during the 4th EW was 2280, Fig. 1. The highest numbers of outbreaks were recorded in Menofia, Dakahlia, Qalyobiya, Fayoum, Gharbia, and Giza governorates respectively, Fig. 2D.

The 5th EW began in October 2012, peaked in March 2013, and ended in June 2013 with no significant outbreak until December 2013 in which 255 outbreaks were reported. The highest numbers of outbreaks were in Menofia, Giza, Gharbia, Behera, and Dakahlia, governorates respectively Fig. 2E. While, the 6th EW began in December 2013, with a significant number (2391) of outbreaks over the whole period until 2017; successive peaks were observed without specific patterns with alteration in the usual epidemic cycles. Unlike all EWs, the highest numbers of outbreaks were in Menia, Sharkia, Suhag, Giza, and Dakahlia governorates respectively, Fig. 2F.

### The spatial pattern of outbreak density

**Fig. 3:**
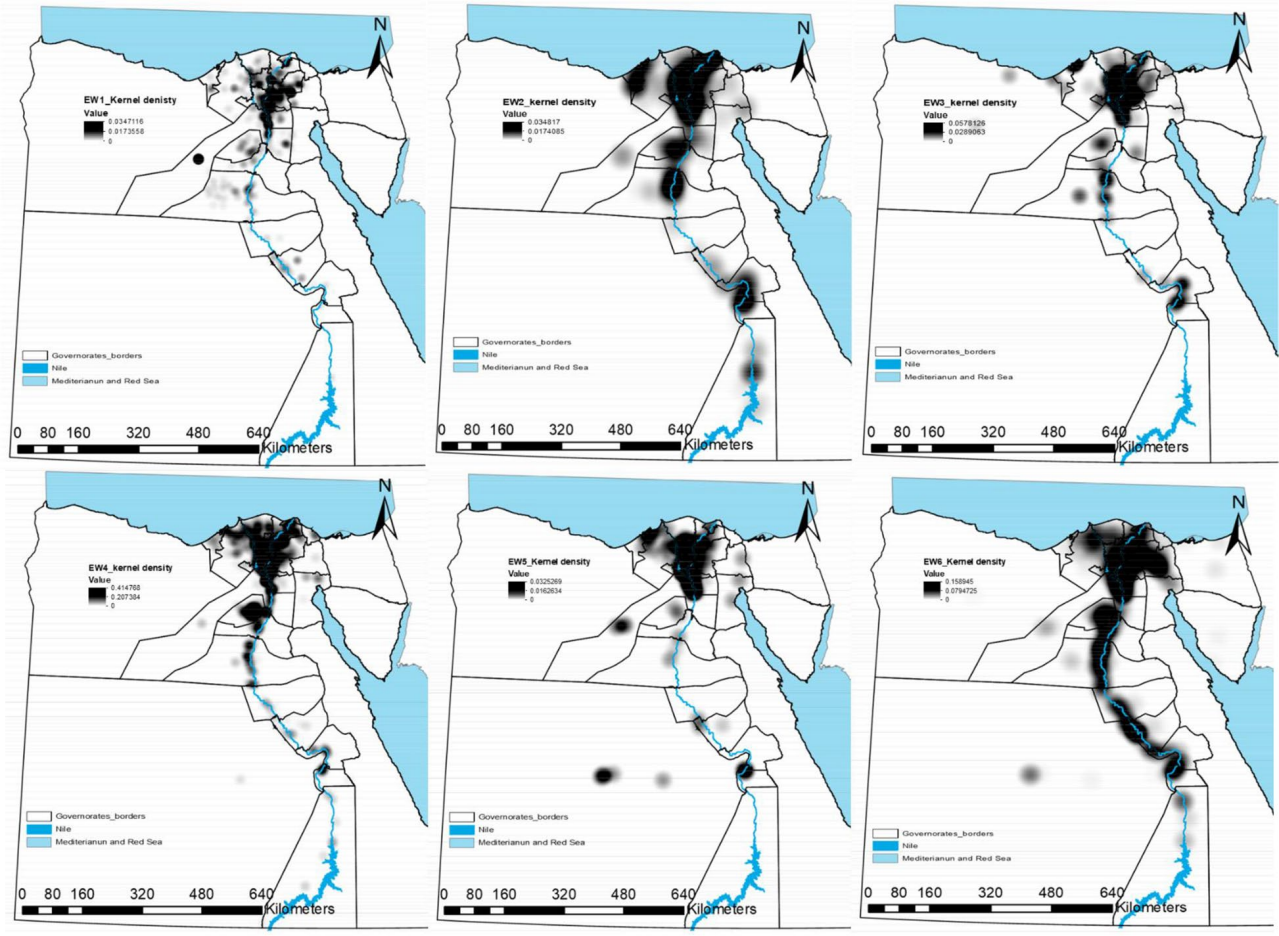
Adaptive kernel density estimation of highly pathogenic avian influenza subtype H5N1 outbreaks in Egypt in six epidemic waves (Highlighted in monochromatic grey: the higher the density, the darker the color)

The outbreaks are represented by black dots and the density from the adaptive kernel density estimation is highlighted in monochromatic grey (the higher the density, the darker the color) and Fig. 3.

The spatial distribution of outbreaks in the 1st EW was confined to the Delta region with low density in Upper Egypt. The highest density was observed in Sharkia, Giza, Qalyobiya, Dakahlia, and Gharbia governorates. In the 2nd EW, outbreak density covers all Delta governorates besides, Alexandria and Damietta with outbreaks beginning to increase toward upper Egypt covering Fayoum, Menia, Qena, Luxor, Aswan with a significant density. The 3rd EW almost has the same distribution as the 2nd EW but with lower sharpness.

In the 4th EW, the outbreaks seem to be connected from Damietta, Behera, Alexandria passing through all delta governorates to Giza, Fayoum, Beni Suef with a lower density in Lower Egypt compared to Upper Egypt. The spatial distribution of outbreaks in the 5th EW was confined to the Delta region with two separate spots of high density in the new valley and Luxor. By the 6th EW, all governorates along the delta region and Nile valley suffered from the highest density of outbreaks.

### Spatiotemporal clusters

**Fig. 4:**
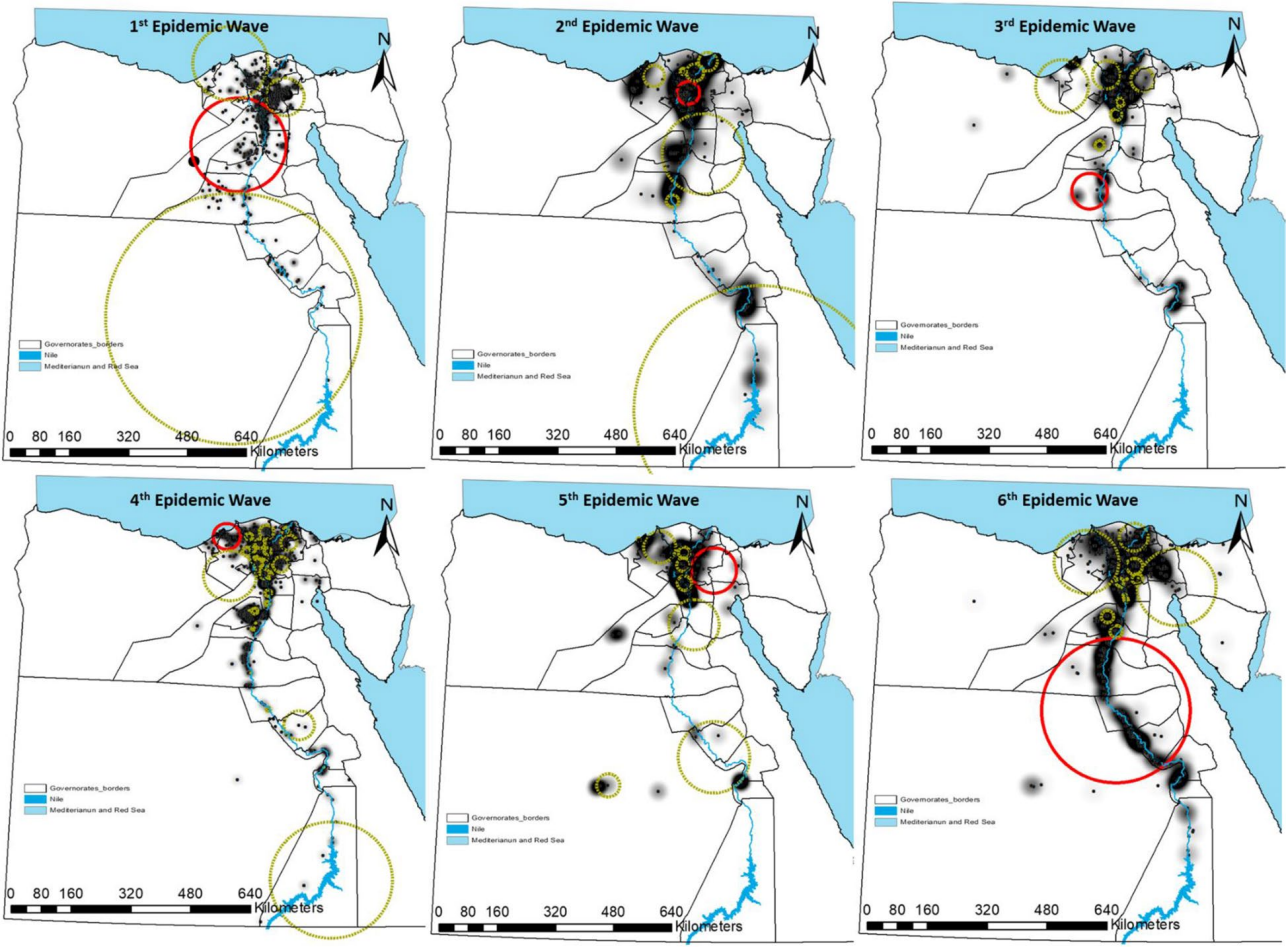
Spatial patterns and Spatiotemporal clusters of weekly outbreaks of highly pathogenic avian influenza subtype H5N1 over six epidemic waves in Egypt. Outbreaks represented by black dots and Outbreak density from adaptive kernel density estimation are highlighted in monochromatic grey (the higher the density, the darker the color). Significant spatiotemporal clusters detected from the space–time permutation scan statistics are illustrated by the most likely cluster (red circle) and by a secondary cluster (light green-dashed circles)

The extent and location of clusters are fully described in (Tables 1 and 2, Fig. 4). The results from daily, weekly, and monthly outbreaks were very similar, therefore only weekly results were reported. Significantly detected Spatiotemporal clusters, from the space-time permutation scan statistics, are illustrated by the most likely cluster (red circle) and by a secondary cluster (light, green-dashed circles) in (Tables 1 and 2, Fig. 4). In the 1st EW, clusters were covering the whole country. The primary clusters with the highest number of locations were stopover sites for wild birds in Fayoum “Lake Qarun, Wadi El Rayan”, and in Behera “Wadi El Naturn”. Also, in Menofia, Qalyobiya, Cairo, Giza, Menia, and Benisuef of 119.7 Km radius. The highest relative risk (RR) clusters were observed in Dakahlia, Qalyobiya, and one cluster in Upper Egypt involving (Menia, Qena, Suhag, Assuit, Luxor, Aswan, New_valley).

**Table 1:**
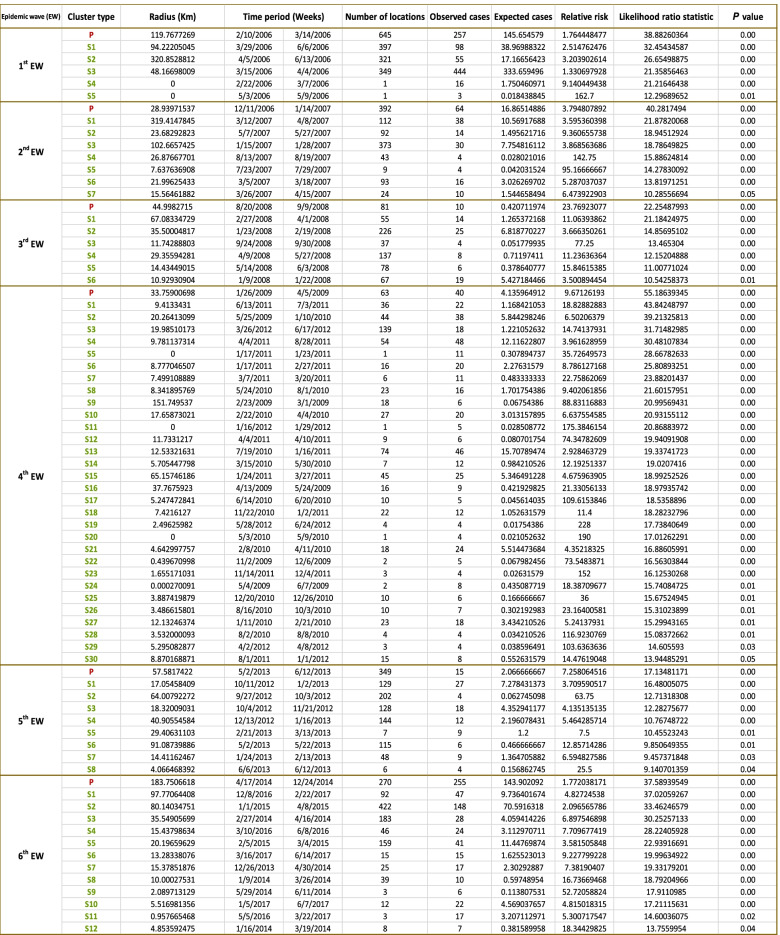
Clusters of HPAI H5N1 outbreaks detected by space-time permutation scan statistic for six epidemic waves in Egypt

^*^P Primary cluster, *S* secondary clusters

**Table 2:**
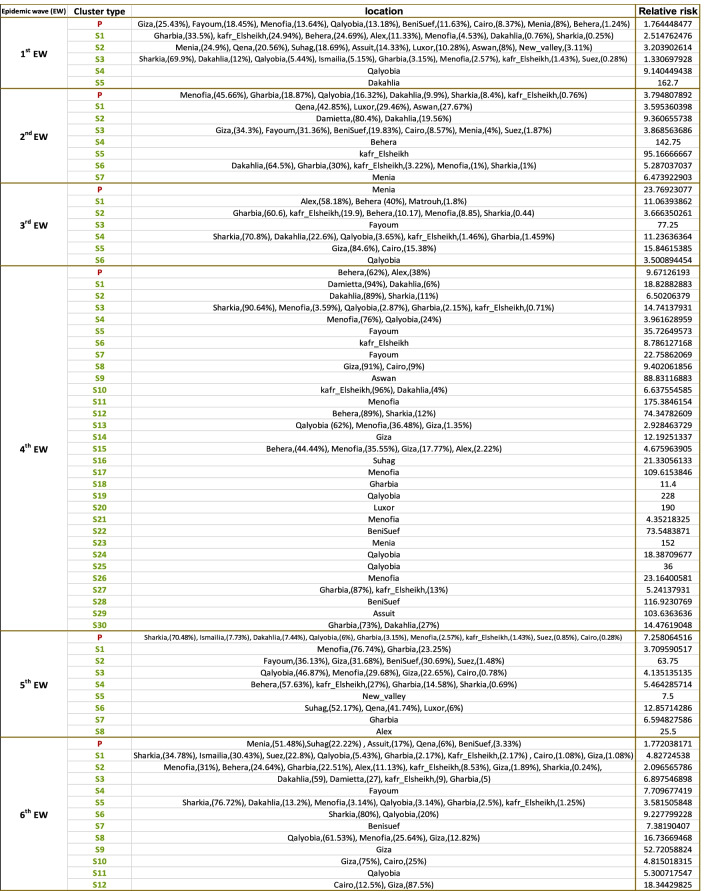
Spatial distribution of significant clusters of HPAI H5N1 outbreaks detected by space-time permutation scan statistic for six epidemic waves in Egypt

^*^P Primary cluster, *S* secondary clusters

In the 2nd EW, the primary cluster of the first outbreak with the highest number of locations and cases of radius 28.9 km was located only in the delta governorates-Menofia, Gharbia, Qalyobiya, Dakahlia, Sharkia, and KafrElsheikh. It is near the migratory bird stopover site “Wadi El Naturn”. Clusters of high RR were also detected in Kafr_Elsheikh, Damietta, Dakahlia, Behera, and Menia governorates (the same governorates appear to have the highest outbreak density).

In the 3rd EW, the primary cluster was detected at the end in the Menia governorate with a 44.9 Km radius. Outbreak clusters continued to appear in the same governorates except for Damietta. The highest RR clusters were in Fayoum, Cairo, Giza governorates. Other clusters with relatively high RR were noticed in the delta region, specifically in Sharkia, Dakahlia, Qalyobiya, Kafr_Elsheikh, Gharbia governorates. It is worth mentioning that no clusters were detected in Upper Egypt.

In the 4th EW, 30 clusters were detected and characterized by small radius size, all confined to the Delta region and Nile valley, with a noticed cluster in Damietta. The primary cluster, the first occurring cluster in the 4th EW, was located in Alexandria and Behera of a 33.7 Km radius. It was including migratory bird stopover sites “Lake Maryut” in Behera and “Lake ldku” in Alexandria. However, lowered density observed in upper Egypt, several clusters in all upper Egypt governorates were detected-unlike the 3rd EW- in Menia, BeniSuef, Assuit, Luxor, Suhag, and Aswan governorates. The highest RR clusters were observed in Qalyobiya, Luxor, Menofia, BeniSuef, Assuit, Aswan, Behera, Sharkia, and Fayoum governorates.

In the 5th EW, newly hot spots with clusters in Ismailia, Suez, and New Vally governorates were detected. With only one cluster conjoin Suhag, Qena, and Luxor governorates of a radius of 91 Km. The primary cluster was detected at the end of the wave of 57.5 Km radius with the highest number of locations in Menofia, Gharbia, Qalyobiya, Dakahlia, Sharkia, Kafr_Elsheikh- besides Cairo, Suez, and Ismailia governorates. It included two migratory bird stopover sites in Ismailia “Bitter Lakes” and Suez. The highest RR clusters were detected in Fayoum, Giza, BeniSuef, Suez, Suhag, Qena, Luxor, and Alexandria governorates.

In the 6th EW, clusters previously detected in Ismailia and Suez continued to be detected with clustering in all delta regions and Nile valley governorates. A special primary dislodged cluster was detected in Menia, Suhag, Assuit, Qena, and BeniSuef. The Primary cluster occurred as the fifth cluster in the first 4 months of the beginning of the wave and lasted 8 months to the end of 2014 with the highest number of cases and of 183.7 Km radius. Clusters of high RR in Giza, Cairo, Qalyobiya, Menofia, Sharkia, Fayoum, Benisuef, Dakahlia, Damietta, Kafr_Elsheikh, and Gharbia governorates were identified.

## Discussion

In Egypt, HPAI H5N1 possesses a severe impact on the poultry industry and constitutes a serious pandemic threat. To provide a better understanding of the characteristics of HPAI H5N1 outbreaks in Egypt, this study aimed to explore the spatiotemporal pattern and identify clusters of HPAI H5N1 outbreaks from 2006 to 2017 as illustrated in (Graphical abstract). The results showed that six EWs over different time scales of daily, weekly, and monthly had similar patterns, which may indicate that HPAI H5N1 has a strong epidemic characteristic. The duration of the EWs (1 to 6) was 8, 15, 12, 46, 13, and 50 months, respectively, and the starting dates were from August to October/November. Together, these findings suggest that the disease control strategies were effective to some extent [51], however, the epidemic cycle was not interrupted. Z Zhang, D Chen, Y Chen, TM Davies, J-P Vaillancourt and W Liu [50] found that the number of HPAI H5N1 outbreaks was decreased over time, and this was indicated by the shorter vertical span of the EC and extensive spatial distribution with a long horizontal span over time. This suggests that there is a potential risk of spatial spread and some of these outbreaks may have been ongoing for a while and the peaks were detected by improved passive surveillance [52].

The EC revealed that outbreaks in each EW started in winter “October, November, or January”, reached the peak in March, and ended in July or August. This suggests a seasonal pattern (winter, and early spring) of HPAI H5N1 outbreaks in poultry with the highest risk period seems to be from October to March. This may be due to a high activity and survival rate of the virus associated with environmental factors of low temperature and high relative humidity during the winter season [17, 33, 53–57]. This Epidemic cycle was observed during the first three EWs (2006–2008) and is in an agreement with previous studies [8, 9, 17, 56, 57]. There were no observed outbreaks in warmer months until the 4th EW with no alteration in the usual epidemic cycle until the beginning of the 6th EW in which there were successive peaks without a specific pattern. This also indicated the environmental impact on the activity and survival rate of the virus.

Clade 2.2.1 was detected during the 1st and 2nd EWs while clade 2.2.1.1. was detected in the 2nd EW. After the disease became endemic in 2008, both clades were existing during the 3rd EW. Isolates of H5N1 viruses showed a rise in genetic diversity in the 2.2.1.2 cluster from early 2008, shortly after the first detection in 2006. During the 4th EW, in addition to clade 2.2.1 and clade 2.2.1.1 that were present during the 3rd EW, clade 2.2.1.1a and 2.2.1.2 were detected and H9N2 was reported for the first time in Egypt. Our results are in good agreement with [4].

Generally, the incidence of HPAI H5N1 decreased throughout the summer and autumn seasons when the temperature increased [1]. However, it was noticed that during the 4th EW in 2009, the virus was circulating all year round with alteration in the epidemic cycle [1, 8, 9, 58]. Several successive peaks in the 4th EW were observed and the wave lasts longer compared to other precedeing waves. This is consistent with [59] observations for the outbreaks in the Menofia governorate. It could be attributed to newly emerged thermostable clades 2.2.1.1 and 2.2.1.2-b/2.2.1.2-c, or the higher ability of the virus to survive longer at higher temperatures (56 °C degrees) [58]. The adaptation of these clades to the Egyptian environment leads to the endemic status of the virus [60]. From 2009 to 2014, the 2.2.1.2 cluster exhibited a constant progressive adaptation to poultry and was considered to be an endemic cluster becoming the dominant cluster circulating since 2011 [4]. Viruses within the variant clusters were less fit than the viruses of the classic clade 2.2.1, ultimately giving rise to a group of endemic clade 2.2.1.2 viruses [60].

By the end of the 4th EW, there was no significant peak in 2012 but there were cases at the same time of the epidemic cycle with no recorded outbreaks from August 2012 to the beginning of the 5th EW; in which the peak was flattened compared to others. The same clades were detected during the 4th, 5th, and 6th EWs (clade 2.2.1, clade 2.2.1.1a, and 2.2.1.2) [4]. The emergence of H9N2, H5N8, and H5N2 in 2011, 2016, and 2019, respectively, have complicated the endemic situation with an EC that ended up without any specific pattern in the 6th EW.

Kernel densities of HPAI H5N1 outbreaks in all EWs revealed distinct outbreak patterns. These different spatial patterns suggest different spread mechanisms [27]. The highest density in all six EWs suggested that Delta was a ‘Hot spot’ for most outbreaks with different locations and sizes. In the last EW, the hot spot emerged to cover all delta and the Nile valley (Fig. 3). There are no geographical barriers or borders between most of the Egyptian governorates, therefore the country appears as a small village or one epidemiological unit. Lower density was observed in Upper Egypt compared to Lower Egypt and this was a common feature from the 1st to the 5th EW. The obtained results are broadly consistent with previous studies which indicated that the incidence of HPAI H5N1 was higher in Lower Egypt than Upper Egypt from 2006 to 2009 in commercial farms, backyards, humans, and the outbreaks were concentrated mostly in the Nile delta [8, 9]. The Nile delta, where the disease is most concentrated, has a very high density of domestic waterfowl, rural human population, and an abundance of water and irrigation networks which are high-risk factors for HPAI [61]. It was noticed that the density of HPAI H5N1 outbreaks in poultry has a positive correlation with human population density and proximity to water canals. Both factors were identified as risk factors for HPAI H5N1 outbreaks across different regions and spatial scales [61]. The high density of the human population is usually associated with high poultry production and/or trade activities, resulting in an increased risk of contact with infected poultry [27]. The location of large cities with high demand for poultry products would increase the chances of disease transmission through poultry trade routes [52]. Poultry production and/or trade near wetlands would increase the chances of infection because of the higher risk of contact with infected domestic waterfowl, infected wild birds, or contaminated environment [27] as in the delta region and the Nile valley. The frequent reoccurrence of disease clusters could be due to a high survival rate of the virus in contaminated water and bird feces [53]. In Romania, similar to our findings, MP Ward, D Maftei, C Apostu and A Suru [62] found that HPAI H5N1 outbreaks in village poultry populations were significantly associated with villages less than 5 km from a river or a stream. This could be attributed to the ability of the HPAI H5N1 virus to survive in water or feces for extended periods up to 207 days at 17 °C or up to 102 days at 28 °C [63], and remains virulent in liquid bird feces for 30–35 days at 4 °C and 7 days at 20 °C [27].

The results of HPAI H5N1 cluster epidemics in Egypt in each EW from 2006 to 2017 could be attributed to many risk factors. This includes differences and variety in agro-ecology, human and animal demographic characteristics, poultry production systems, and wild bird’s stopover sites and their habitats. Climate variability has been proved to influence the outbreak occurrence and spread of the virus in the environment [59].

In the 1st EW, the clusters covered almost the whole country and this was consistent with A Arafa, I El-Masry, S Kholosy, MK Hassan, G Dauphin, J Lubroth and YJ Makonnen [60] who reported that H5N1 outbreaks covered 96.3% of the country. Although it is hard to trace the most likely route of introduction from the pattern of spread alone [52], the statistical phylogeography metrics suggest that H5N1 diffusion is geographically structured in Egypt [64].

It has been found that clustering in the Delta was the common feature in all EWs, but the location and size varied. The primary Spatiotemporal cluster was consistently detected in the hot-spot region across all six EWs, but the location and size varied. These results concur with other studies which have shown that the majority of routes between governorates were found in the heavily populated Delta region as a popular location for virus transition [64]. The highest density of poultry population (1000/km^2^) along with human population density in the Nile delta region could be the main reason for the establishment of the virus and clustering in that region [1].

The highest RR clusters in all EWs were found in Sharkia, Gharbia, Fayoum, and Qalyobiya governorates. Our findings suggest that the highest risk regions of the highest RR clusters in all EWs were found in Fayoum, Dakahlia, Qalyobiya, Sharkia, Kafr_Elsheikh, Giza, Behera, Menia, BeniSuef, Luxor governorates. Our results support that the virus spread route was from Sharkia to Gharbia and from Fayoum to Qalyobiya as suggested by [64]. These regions with the highest risk of outbreak clustering have an increased chance of a repeated events than others. In most cases, the primary clusters of next waves were detected in the regions of the highest RR in the previous one. JH Mu, BA McCarl, X Wu and L Gan [65] found a positive significant effect between past and current outbreaks.

The results showed clusters in the cities with zero radii, which increase the possibility of viral spread in the surrounding areas from this point. Cities are characterized by highly intense poultry trade activities including live poultry markets, food markets, slaughterhouses, and poultry processing plants. HPAI virus could be spread through the road networks [66–68] this may also indicate the spread of the disease through the transportation of poultry and poultry products [69]. A high risk of HPAI H5N1 was strongly associated with highly-populated areas, short distances to the highway junction (< 20 km), and a high density of roads since highway junctions considered as “dissemination nodes” for the HPAI H5N1 virus [66–68].

The primary cluster occurred mostly at the beginning of the EW except for the 3rd and the 5th ones. While the secondary clusters appeared both in the early and late periods of each EW (Fig. 4) and varied in number and location over space and time. The clustering of neighboring outbreaks is a common feature in all secondary clusters along EWs from the 3rd to the 6th. The distance between clusters was less than 20 km and has a time interval of less than 3 weeks. From these results, it could be deduced that the transmission of HPAI H5N1 from one location to another directly resulted in a series of outbreaks forming neighboring clusters without efficient intervention to break these chain events. In China, six clusters of HPAI H5N1 in 30 outbreaks and 20 km distance were identified [68]. The subsequent spread of infection in multiple secondary cluster patterns suggests there was an infection reservoir in which the disease was circulating and undetected. Under these scenarios, it is difficult to trace the most likely route of the introduction of infection [52].

This clustering could also be attributed to the high population density of small commercial farms and backyard poultry in Egypt (FAO sectors 3 and s 4) [70]. These sectors suffer from low vaccination, surveillance coverage, and low biosecurity practices.

It was noticed that the primary clusters included stopover sites for migratory wild birds except the 3rd and the 6th EWs. Backyard poultry is a common practice allowing interactions between wild and domesticated birds throughout Egypt [71]. The distribution of the human population combined with the proximity to water canals and/or wetlands is an important interface between poultry and wild birds [27]. During avian fall migration (August–November), bird hunting is most extensive in northern Egypt, Nile Delta, and its surroundings in which wild birds are trapped and traded at local or regional markets [1, 72]. Samples from live bird markets indicated that apparently healthy wild birds were positive for HPAI viruses [3, 73].

The hot spot identified at the border between Europe and Africa covers the Black and Mediterranean Seas and neighboring regions such as Egypt, Greece, and Turkey, was emerged in the 3rd global EW in June 2005 and persisted up until the global 6th EW [50]. Extensive wetlands in this region have formed significant breeding and congregation sites for domestic, migratory, and other wild birds, increasing the risk of contact and facilitating virus evolution [74]. In addition to the existence of two bird migratory routes across this region (the Black Sea–Mediterranean and the East Africa–West Asia flyways), which link Asia, Europe, and Africa [50].

## Conclusion

It was clear that HPAI H5N1 outbreaks usually started in winter, peaked in March, and ended in summer. After the emerging of newly thermostable clades (2.2.1.1 and 2.2.1.2), there was a clear shift in the pattern of the epidemic cycle and the situation became more complicated. During the 4th EW the virus had the ability to survive and cause infection in warmer months with a clear alteration in the usuall seasonal epidemic cycle in the 5th EW. The endemic situation became more complicated by the emergence of new AI serotypes. As a result, the EC ended up without any specific pattern in the 6th EW and till now. The spread of infection was probably taking place at many different but interlinked patterns affected by the density of poultry and human populations, transportation of poultry and their products; infected reservoirs; proximity to rivers or wetlands; and wild bird hunting and trade.

The spatial distribution indicated that the spread of the HPAI H5N1 is probably taking place at many different but interlinked patterns. The spread patterns responsible for local transmission are the following: highest density of poultry population along with human population density; transportation of poultry and their products; the transmission of HPAIH5N1 occurred from one location to another directly resulted in a series of outbreaks forming neighboring clusters without efficient intervention; disease circulated undetected through infected reservoir; proximity to rivers or wetlands is an important interaction gate between poultry and wild birds; and, wild bird hunting and trade in LBM. They could exist simultaneously together as in the Nile delta and along Nile valley. The dynamics of how the virus survives are important for a country’s decision of whether to implement disease prevention and control strategies. One of the limitations of our study is that the results are largely dependent on the quality of the original data. In addition to the common problem of underreporting due to fear of culling and inadequate compensation, under the coverage of surveillance program and the sensitivity of the active surveillance is not enough to declare sporadic occurrence or areas that are free from infection. Our findings can help in better understanding of the characteristics of HPAI H5N1 outbreaks and the distribution of outbreak risk, which can be used for effective disease control strategies.

### Study limitations

One of the limitations of this study is that the results are largely dependent on the quality of the original data. In addition to underreporting due to fear of culling and inadequate compensation. The sensitivity of the active surveillance is not enough to declare sporadic occurrences or areas that are free from infection. Detection bias, control measures, or changes in demographic characteristics in at-risk populations couldn’t be considered.

## Data Availability

All data generated or analyzed during this study are included in this published article.
